# Current Standards and Recent Advances in Biomarkers of Major Endocrine Tumors

**DOI:** 10.3389/fphar.2018.00963

**Published:** 2018-09-10

**Authors:** Yanhong Luo, Hua Zhu, Tao Tan, Jianfeng He

**Affiliations:** ^1^Children’s Hospital of Chongqing Medical University, Chongqing, China; ^2^Department of Surgery, Davis Heart and Lung Research Institute, The Ohio State University Wexner Medical Center, Columbus, OH, United States

**Keywords:** biomarkers, thyroid carcinoma, neuroendocrine carcinoma, pituitary carcinoma, adrenal carcinoma

## Abstract

The complexity of endocrine tumor diagnosis stems from its variable symptoms and presentation that may mimic many other disease states, or display asymptomatic properties for a prolonged amount of time. Early and accurate disease identification is needed for better patient prognosis. The key to this may be in using validated biomarkers with enhanced sensitivity and specificity. Several biomarkers are consistently used across various endocrine tumor types, possibly indicating a deeper pathophysiological mechanism behind endocrine cancer genesis and development. For example, carbohydrate antigen (CA) is measured in both pancreatic adenocarcinoma as well as ovarian cancer for diagnosis, surveillance, and risk stratification. The discovery of measuring miRNAs that are highly expressed in malignant tumors is also a novel strategy across multiple endocrine tumor types, and is propelling the future advancement of biomarker development. This review introduces currently utilized biomarkers in some of the commonly known endocrine tumors, including thyroid, adrenal, pituitary, pancreatic, and gonadal carcinoma, as well as future research directions.

## Introduction

Endocrinology comprises of a complex web of hormonal regulations. Understanding the intricacies of glands and hormones produced, as well as their downstream effects, is critical to the success of the field of endocrinology. It is difficult to categorize endocrinology strictly by organ systems, as its impact falls across multiple anatomic areas. On the other hand, the management of endocrinopathology is very simply summarized by initially assuming that most endocrine disorders can be treated by hormone replacement or suppression when accurately determined (Larry Jameson, 2015). However, variable symptoms and presentation that may mimic other disease states can make initial diagnosis difficult (Wolin et al., 2017). The key to correct disease identification may be in using biomarkers. Particularly for malignant tumors that require immediate medical attention, it is vital that clinically verified biomarkers be developed and utilized for timely diagnosis, and subsequently, to track treatment progress.

The current standard across most endocrine tumors includes an initial workup of measuring the level of offending hormone; yet this is generally low in specificity as abnormal hormone levels can also indicate other non-tumor disorders. Further biomarkers found in the serum and imaging diagnostics are required to confidently pinpoint the cancer diagnosis and monitor treatment success. However, many of these have not been shown to provide the specificity and sensitivity required for accurate surveillance. With the advancement of biotechnologies, scientists may be turning increasingly to newer biomarkers such as micro RNAs (miRNAs). In this review, we discuss some of the most well-known endocrine tumors, including thyroid, adrenal, pituitary, pancreatic, and gonadal carcinoma, and their associated biomarkers, as well as future research directions.

## Thyroid Carcinoma

Thyroid carcinoma is the malignant tumor of thyroid epithelia, which is also known as thyroid nodules. Thyroid carcinoma occurs infrequently when compared with the incidence of thyroid nodules. The lifetime diagnosis risk of thyroid carcinoma is 1.2% (Noone et al., 2018). The prevalence of thyroid nodules is currently about 5%, and the probability of developing thyroid nodules increases with older age (Noone et al., 2018). If tumors are present, identifying between aggressive and benign tumors can be a major challenge in thyroid pathology (Baloch et al., 2018). Thyroid carcinomas are categorized into differentiated (e.g., papillary, follicular, and Hürthle cell), medullary, and undifferentiated or anaplastic tumors (The National Comprehensive Cancer Network [NCCN], 2018e).

### Diagnosis of Thyroid Carcinoma

Because both benign and malignant nodules can remain asymptomatic, diagnosis is often delayed and can lead to worse prognosis (The National Comprehensive Cancer Network [NCCN], 2018e). The protocol for evaluating thyroid carcinoma is well established. First and foremost, the thyroid-stimulating hormone (TSH) level is measured, and an ultrasound is performed to determine whether a fine-needle aspiration (FNA) is necessary (Czerwonka et al., 2014). Ultrasound features are often interpreted with high observer variability. As such, further tests may be required to confirm the diagnosis. Serum thyroglobulin (TG) levels can be taken, but are elevated in most thyroid diseases and may be relatively non-specific to tumors. Serum calcitonin levels can help with the early detection of medullary thyroid cancer (MTC) or tumors of C-cell origin. Focal (^18^F) fluorodeoxyglucose positron emission tomography (^18^FDG-PET) imaging is being increasingly utilized in malignant and non-malignant disease evaluation (Haugen et al., 2016). Soelberg et al. reported in a meta-analysis that approximately 35% of focal ^18^FDG-PET positive thyroid nodules were cancerous (Soelberg et al., 2012). Contrastingly, diffuse ^18^FDG-PET uptake is associated with benign disease. This test should be combined with sonographic evidence for further clinical confirmation. It is recommended that FNA cytology interpretation be standardized to the Bethesda System for Reporting Thyroid Cytopathology, which consists of six categories of malignancy risk (Crippa et al., 2010).

### Molecular Testing of Thyroid Carcinoma

Recent advancement in molecular technology has made it possible to more conveniently test the molecular biomarkers in thyroid carcinoma. The most common molecular biomarkers are involved in the analysis of gene mutation profile, epigenetic profile, microRNA profile, and cancer stem cell biomarkers. Molecular testing of FNA samples can further specify patient diagnosis, prognosis, therapeutic benefit, or aid in active observation (The National Comprehensive Cancer Network [NCCN], 2018e). Immunohistochemistry may be supplemental in confirming and differentiating thyroid tumors from other endocrine cancers, to further sub-classify tumor cell types or high-risk mutations. These biomarkers are critical in guiding individualized cancer treatment (Baloch et al., 2018). Most commonly studied genetic biomarkers include a seven-gene panel of mutations (*BRAF, RAS, RET/PTC*, and *PAX8/PPARγ*) (Nikiforov et al., 2011), a gene expression classifier (mRNA expression of 167 genes) (Alexander et al., 2012), and galectin-3 immunohistochemistry (Bartolazzi et al., 2008). These tests have been shown to rule out malignancy in indeterminate cytology specimen or to guide surgical procedures. A variety of molecular marker test are also available in hospital-based laboratories for the ease of clinician.

### Common Biomarkers in Thyroid Carcinoma

The biomarkers for thyroid carcinoma are numerous and diverse; therefore it is important to understand the nuances in biochemical and imaging test results to appropriately identify tumor types. For example, the human TG levels, though a rather insensitive form of measurement, can be significantly increased in patients with follicular-derived thyroid cancers as compared with those with benign conditions (Baloch et al., 2018). TG expression can give a clue to the thyroid follicular origin of the tumor, or can be decreased in the case of poorly differentiated thyroid carcinomas. Additional examination can reveal dense granular deposits localized to the perinuclear area or some cases of weak and focal expression in the case of oncocytic (Hürthle cell) tumors. Huürthle cell carcinoma can also look similar to medullary carcinoma on a frozen section (The National Comprehensive Cancer Network [NCCN], 2018e). Rare thyroid tumors can express both TG and calcitonin in the follicular cell- and C-cell-derived constituents, respectively. Other tests may be required to confirm the diagnosis (Baloch et al., 2018). On the imaging side, gallium-68 (^68^Ga) radiolabelled somatostatin analog peptides in PET/CT have been well established for the diagnosis of neuroendocrine tumors. A study by Castroneves et al. showed that ^68^Ga PET/CT may also be highly sensitive to identifying bone metastases in thyroid carcinoma (Castroneves et al., 2018).

### Future Directions of Thyroid Carcinoma Biomarkers

Future research looks to optimize these molecular biomarkers for determining the diagnosis, prognosis, and treatment success. For example, mRNA (Lappinga et al., 2010) and miRNA (Agretti et al., 2012) markers have shown promise in diagnostic utility but have yet to be validated. Agretti et al. used data mining to identify miRNA with predictive enough expression to differentiate between benign and malignant nodule. They found that miR-146b, miR-187, and miR-224 expressions were significantly increased in papillary thyroid carcinoma compared with in benign nodules (Agretti et al., 2012). Peripheral blood TSH receptor mRNA assay has been associated with high positive predictive value (90%) and negative predictive value (39%) in a single-center, prospective study (Milas et al., 2010). Longer-term studies are required to standardize and determine the validity of the diverse options for molecular testing.

## Neuroendocrine and Adrenal Carcinomas

Neuroendocrine carcinomas result from cells throughout the diffuse endocrine system (The National Comprehensive Cancer Network [NCCN], 2018a). Most common of these are well differentiated or carcinoid tumors, which are gastroenterohepatic and pulmonary in origin and exhibit a variety of symptoms (Gabriel et al., 2018). Patients may present with symptoms associated with hormonal hypersecretion (“functional” tumors) or those that are asymptomatic are considered to have “non-functional” disease (The National Comprehensive Cancer Network [NCCN], 2018a). Furthermore, due to the sporadic and gradual nature of tumor growth, as well as their small size, the lesions can be difficult to detect (Gabriel et al., 2018).

The most common inherited syndrome associated with neuroendocrine tumors is multiple endocrine neoplasia type (MEN) 1 (Oberg, 2013). Inherited genetic syndromes such as MEN types 1 and 2 are characterized by tumors of a number of origins depending on mutation type. For example, in MEN type 1, mutations in *menin* gene is associated with tumors of parathyroid, pituitary, and pancreatic glands, while MEN 2 is characterized by mutations in the *RET* proto-oncogene and leads to the development of medullary thyroid carcinoma, pheochromocytoma, and hyperparathyroidism (The National Comprehensive Cancer Network [NCCN], 2018a). Other genetic aberrations found upon mutational analysis include the VHL gene in pancreatic neuroendocrine tumors (NETs) and JAK3, NRAS, RB1, and VHL1 in pulmonary NETs (Oronsky et al., 2017).

### Diagnosis of Neuroendocrine Carcinomas

Neuroendocrine carcinomas are histologically categorized by tumor differentiation (The National Comprehensive Cancer Network [NCCN], 2018a). Generally, tumor grade is positively correlated with mitotic count and Ki-67 proliferation index, while the classification method differs depending on tumor origin (Klimstra et al., 2010). Staging is according to the AJCC tumor, node, metastasis (TNM) system and determined similarly to other cancers of organs in its physiologic vicinity (Edge et al., 2010). In other words, for example, carcinoids of the lung and bronchi are staged in the same manner as common lung carcinomas, and carcinoids of gastrointestinal origin are staged by other means.

Various imaging techniques are important in initial detection and evaluating the tumor grade of NETs. The current standard work-up includes CT and MRI; clinicians may additionally consider using somatostatin receptor scintigraphy (SRS) (The National Comprehensive Cancer Network [NCCN], 2018a). One of the standard imaging techniques utilizes ^111^Indium-diethylenetriaminepentaacetic acid (^111^In-DPTA)-octreotide. Newer, more sensitive PET radiotracers, such as ^18^F-fluorodopa and ^68^Ga-labeled somatostatin analogs are also utilized for more differentiated tumors (Gabriel et al., 2018).

### Common Biomarkers of Neuroendocrine Carcinomas

One commonly used protein marker is chromogranin A (CgA), which is elevated and particularly useful in patients with non-functioning neuroendocrine tumors as well as in its ability to indicate poorer prognosis (Kapoor et al., 2014; Oronsky et al., 2017). Evaluating serotonin secretion using a 24-h urine collection of a serotonin metabolite, 5-Hydroxyindoleacetic acid (5-HIAA), is another biomarker assessment recommended for patients with metastatic lung or GI carcinoid tumors (Kapoor et al., 2014; Oronsky et al., 2017). Diet and drugs can affect 5-HIAA levels, however, and therefore proper patient counseling is warranted before this test (The National Comprehensive Cancer Network [NCCN], 2018a). Another study found that mammalian target of rapamycin (mTOR) overexpression correlated with decreased overall survival (OS) (Qian et al., 2013). Mutations in cyclin-dependent kinase inhibitor, CDKN1B (p27) or loss of CDKN1B expression have been observed in gastroenteropancreatic neuroendocrine tumors (Kim et al., 2014). Khan et al. (2013) hypothesized that circulating tumor cells (CTC) would suggest a more disseminated disease and found that the presence of CTC was associated with decreased progression-free survival and OS.

### Future Directions of Neuroendocrine Carcinoma Biomarkers

Because of the heterogeneity of the disease, neuroendocrine neoplasms secrete a vast spectrum of protein or hormonal markers aside from CgA and 5-HIAA, such as insulin, gastrin, glucagon, somatostatin, growth hormone, calcitonin, substance P, pancreastatin, etc (Oronsky et al., 2017). This makes detection even more difficult for an already rare disease. Having a common biomarker may ease the process and facilitate earlier tumor diagnosis, as pursued, for example, by Gabriel and colleagues, who demonstrated that using ^68^Ga-DOTATATE PET/CT for primary detection was superior than standard work-up (Gabriel et al., 2018). Yet, there is still a dearth of knowledge on the molecular basis of neuroendocrine tumors, and therefore more studies are needed to validate these molecular assays for clinical application. To date, no single biomarker has been accepted for use in neuroendocrine tumors (Oberg et al., 2015).

## Pituitary Adenocarcinomas

Pituitary tumors often present as sellar masses. Seller masses can form as frequently as in about 10–15% of the adult population. In contrast, pituitary carcinomas (PCs) are one of the most rare but aggressive tumor types, representing only 0.1–0.2% of all pituitary tumors (Ragel and Couldwell, 2004).

### Diagnosis of Pituitary Carcinoma

Pituitary carcinomas often present as invasive macroadenomas that metastasize systemically (Ragel and Couldwell, 2004). MRI is used to diagnose the extent of sellar masses (Ragel and Couldwell, 2004). In particular, imaging techniques that accurately measure the dimensions and invasions of the tumor site are recommended (Raverot et al., 2018). For example, SRS with ^111^In-DPTA-octreotide can identify distant pituitary metastases (Ragel and Couldwell, 2004). Consistent tumor measurements are necessary to track tumor progression and guide treatment throughout the course of the disease (Raverot et al., 2018).

### Common Biomarkers of Pituitary Carcinoma

Pituitary carcinomas harbor more commonly renowned neoplastic biomarkers such as Ki-67 and p53. Since the main characteristic of PCs is the mitoses of tumor cells, histopathological results often show an increase in MIB-1 staining index (for Ki-67) as well as a higher level of p53 protein (Ragel and Couldwell, 2004; Raverot et al., 2018). Immunohistochemical findings are often used as differential diagnosis from other diseases, such as Cushing’s syndrome or other metastatic tumors (Hirohata et al., 2014). Majority of PCs are hormone-secreting tumors, including adrenocorticotropic hormone (ACTH) secreting and prolactin (PRL) secreting tumors (Hirohata et al., 2014), and as such, antibodies for these hormones are used to aid in the diagnosis of pituitary tumor origin (Ragel and Couldwell, 2004). Other biomarkers that help identify tumor source are cytokeratin, epithelial membrane antigen, glial fibrillary acidic protein (GFAP), and CgA (Ragel and Couldwell, 2004).

### Molecular Testing of Pituitary Carcinoma

Molecular and cytogenetic determination of PCs may play a role in better characterizing PCs. The *Gsp* gene is present in about 40% of growth hormone (GH)-producing tumors and the *Ras* oncogene is associated with anaplastic progression (Ragel and Couldwell, 2004). Higher level of p53 is indicative of an aggressive course of disease. A quantitative immunohistochemical study showed that p53 is expressed in 0 and 100% of non-invasive and metastatic disease, respectively (Ragel and Couldwell, 2004).

### Future Directions of Pituitary Carcinoma Biomarkers

Early diagnosis of PC is challenging but extremely important due to the morbidity associated with the disease (Raverot et al., 2018). Despite advances in research, however, a single biomarker has yet to be found that accurately predicts tumor behavior (Raverot et al., 2018). Other observed molecular or cytogenetic markers that could be potential biomarkers includes the *H-ras* gene mutations, the presence or absence of D2 receptors, measured telomerase activity via hTERT expression, the expression of HER-2/neu, the detecting of cyclooxygenase-2 (COX-2) enzymes, and the involvement of the *Rb* gene (Ragel and Couldwell, 2004; Sav et al., 2012). For detecting high risk disease, FGFR4, MMP, PTTG, and deletions in chromosome 11 in addition to identifying levels of Ki-67 and p53 have been suggested as concerns for aggressive disease management (Mete et al., 2012). Finally, there is a dire research need for standardized criteria for evaluating biomarkers for PCs to validate them as having predictive value (Sav et al., 2012).

## Pancreatic Adenocarcinomas

Pancreatic adenocarcinoma is the fourth most common cause of death in the United States (Siegel et al., 2017). Detection of pancreatic cancer is a challenge because early signs of pancreatic cancer are rarely seen (The National Comprehensive Cancer Network [NCCN], 2018c). Tumor presence is often predicted from astute observation of patient medical histories and presenting symptoms. Therefore, there is a strong consensus on the importance of developing biomarkers for the early diagnosis of pancreatic cancer to prevent further morbidity and mortality. Tumor-associated antigens that are related to pancreatic adenocarcinoma are numerous and include those such as carcinoembryonic antigen (CEA), pancreatic anti-oncofetal antigen, tissue polypeptide antigen, carbohydrate antigen (CA) 125, and CA 19-9 (The National Comprehensive Cancer Network [NCCN], 2018c).

### Diagnosis of Pancreatic Adenocarcinoma

The primary means of diagnosis of pancreatic adenocarcinoma is through imaging. All patients with suspected disease should undergo initial CT evaluation, and continue imaging after every step of therapy (e.g., after neoadjuvant treatment), which provides constant assessment. The TNM staging criteria by the AJCC is utilized to determine whether the tumor is resectable (Edge et al., 2010). The initial recommendation is to screen with endoscopic ultrasounds (EUS) and/or MRI for high-risk individuals (i.e., those with cancer in the family or those that are carriers of *p16* or *BRCA2* mutations) (The National Comprehensive Cancer Network [NCCN], 2018c). A biopsy enhances the pathologic diagnosis of adenocarcinoma before the administration of neoadjuvant therapy (Brugge et al., 2014). Fine-needle aspiration (FNA) guided by EUS is preferred (Brugge et al., 2014). Molecular analyses may supplement EUS by observing for some of the most common mutations in pancreatic cancer, including *KRAS, TP53, CDKN2A*, and *SMAD4* (Waddell et al., 2015).

### Common Biomarkers of Pancreatic Adenocarcinoma

Carbohydrate antigen 19-9 is currently the most validated biomarker for early diagnosis and surveillance of pancreatic cancer. However, it is not tumor-specific, as CA 19-9 is often also expressed in other pancreatic diseases, other malignancies, as well as hepatobiliary disease (The National Comprehensive Cancer Network [NCCN], 2018c); nevertheless, it is still useful in differentiating from inflammatory conditions (The National Comprehensive Cancer Network [NCCN], 2018c). CA 19-9 has a sensitivity of 79–81% and a specificity of 80–90% in symptomatic patients (Huang and Liu, 2014). It is also beneficial to evaluate CA 19-9 as a prognostic biomarker, since a low level or a decreasing level were associated with better median survival in patients with resectable disease (Humphris et al., 2012; Bauer et al., 2013).

Furthermore, CA 19-9 may guide treatment in post-operative settings. In a study of 260 patients, those with CA 19-9 levels less than 90 U/mL benefited from adjuvant therapy by showing a longer disease-free survival (DFS), while those with higher CA 19-9 levels receiving adjuvant therapy showed no difference in DFS from untreated patients (Humphris et al., 2012). This prognostic benefit is also reflected in neoadjuvant or borderline resectable disease (Tzeng et al., 2014) and advanced disease (Bauer et al., 2013; Pelzer et al., 2013), as demonstrated by longer OS correlating with lower CA 19-9 levels. The NCCN guidelines recommend drawing serum CA 19-9 levels before and immediately following surgery, before administration of adjuvant therapy, and for surveillance (The National Comprehensive Cancer Network [NCCN], 2018c).

### Molecular Testing of Pancreatic Adenocarcinoma

Novel biomarkers to screen for early pancreatic cancer are currently being investigated. Notably, there is much improvement in technology that identifies microRNAs in whole blood, profiles serum metabolism, and measures circulating cell-free DNA as possible biomarkers for screening (Schultz et al., 2014). Additionally, CA 19-9 (which is currently used as part of diagnostic testing) was also found to be elevated in pancreatic patients even before the cancer diagnosis, and therefore could be a potential biomarker for screening high-risk patients and for measuring response to chemotherapy (Morris-Stiff and Taylor, 2012). Another study, a meta-analysis, concluded that S100 calcium-binding protein P (S100P) shows high sensitivity (0.87; 95% CI, 0.83–0.90) and specificity (0.88; 95% CI, 0.82–0.93) for pancreatic cancer diagnosis (Hu et al., 2014).

### Future Directions of Pancreatic Adenocarcinoma Biomarkers

While surgical removal of pancreatic cancer can result in better survival, only about 15 to 20% of patients still have localized disease at the time of diagnosis. Therefore, researchers are furiously validating biomarker candidates for earlier detection of disease. For example, Capello et al. (2017) demonstrated the validation of a panel of immunoassays as biomarker candidates, and found that TIMP1 and LRG1 in combination with CA 19-9 enhanced the detection of early-stage pancreatic cancer when compared with CA 19-9 alone. More evidence is needed for the optimal diagnosis and management of pancreatic lesions. Global experts are urging clinicians to share tissue samples to facilitate the research of potential diagnostic and prognostic biomarkers for pancreatic adenocarcinoma (The National Comprehensive Cancer Network [NCCN], 2018c).

## Gonadal Adenocarcinomas – Testicular Cancer and Ovarian Cancer

### Testicular Cancer, Its Diagnostic Biomarkers and Future Directions

Within all testicular cancer cases, 95% of them consist of germ cell tumors (GCTs) (The National Comprehensive Cancer Network [NCCN], 2018d). Biomarkers that aid in the diagnosis, prognosis, and to monitor treatment are well recognized in this disease state. Classic serum markers utilized for GCTs are alpha-fetoprotein (AFP), lactate dehydrogenase (LDH), and beta-human chorionic gonadotropin (beta-hCG) (Gilligan et al., 2010). Beta-hCG is often the most elevated serum biomarker in testicular cancer and its levels differ depending on the disease type (i.e., seminomatous and non-seminomatous) and metastatic risk, making it a useful diagnostic tool (Ferraro et al., 2018). Minor changes in beta-hCG should be interpreted cautiously, however, as it could be confounded with other conditions (Gilligan et al., 2010; Ferraro et al., 2018). Increased AFP levels are often associated with non-seminomatous disease and can be detected at any stage, but must also be analyzed with care; AFP levels that rise steadily can indicate metastasis, but clinicians may hold initial treatment in patients with only mildly increased and stable AFP levels (Gilligan et al., 2010). Another biomarker, LDH, is utilized in the risk-stratification of patients who are starting initial chemotherapy (Gilligan et al., 2010). LDH may be measured to check for relapse, but it is noteworthy that LDH is relatively non-specific with a high false-positive rate and should not be used alone to indicate treatment (Gilligan et al., 2010). These biomarkers guide clinicians in selecting further diagnostic tests (e.g., PET scan or MRI) and in choosing the appropriate course of treatment.

Testicular cancer is staged using the AJCC TNM system and based on the beta-hCG, LDH, and AFP levels (The National Comprehensive Cancer Network [NCCN], 2018d). Additionally, an ultrasound can complement diagnosis by imaging testicular mass and its surrounding conditions. Treatment is mainly radical orchiectomy, and chemotherapy as required. After the procedure, serum biomarkers will be continuously monitored for their physiological kinetics to determine whether the levels are decreasing at target rates. A slower decline than expected can suggest metastatic disease (The National Comprehensive Cancer Network [NCCN], 2018d).

Despite the standardized approach to measuring beta-hCG, AFP, and LDH levels, these traditional serum biomarkers may not be elevated in a significant number of GCT patients (Looijenga et al., 2014). Therefore, other biomarker candidates have been suggested, such as analyzing the expression of *XIST* gene and detection of specific miRNAs such as from the miR-371-3 and miR-302/367 clusters (Looijenga et al., 2014; Mir et al., 2016). Recently, a number of newer protein markers have been identified to differentiate between histologic subtypes of testicular cancer, such as High Mobility Group A (HMGA), POZ-AT hook-zinc finger protein (PATZ), Aurora-B, Nek-2, c-kit, PLAP, NANOG, SOX2, and CDK10 (Mir et al., 2016). *In vitro* research shows varying levels of overexpression based on tumor differentiation (i.e., seminoma, embryonic carcinoma, or yolk cell tumors). Study of these biomarkers may hold promise for additional utility for diagnosis or active surveillance of testicular cancer (Mir et al., 2016).

### Ovarian Cancer, Its Diagnostic Biomarkers and Future Directions

In contrast to testicular cancers, ovarian tumors consist of a more diverse collection of histopathologic entities (Kurman et al., 2014). Majority of ovarian cancer cases comprise of the epithelial type; others include germ cell tumors and sex-cord stromal cell tumors, as well as a number of rare pathological types (Prat, 2012). The difficulty in treating ovarian cancer mainly stems from the difference in therapy based on each tumor type (The National Comprehensive Cancer Network [NCCN], 2018b). As such, in the United States, it is the fifth most common cause of cancer death in women (The National Comprehensive Cancer Network [NCCN], 2018b).

Due to the physiological location of the ovaries, screening is extremely difficult at an earlier stage (The National Comprehensive Cancer Network [NCCN], 2018b). Therefore, it may be pertinent to find serum biomarkers that help detect less mature disease. While routine screening is not currently required in all women, clinicians may monitor high-risk patients by measuring cancer antigen 125 (CA-125) and performing endovaginal ultrasounds (Smith et al., 2015).

There are multivariate index assay tests previously used for ovarian cancer screening that may now be outdated (Ueland, 2017). Several professional health organizations are now stating that the OVA1 test (which uses five markers: transthyretin, apolipoprotein A1, transferrin, beta-2 microglobulin, and CA-125) should not be used to assess whether patients are candidates for surgery (Timmerman et al., 2016). Additionally, the OvaSure screening test (which uses six biomarkers: leptin, prolactin, osteopontin, insulin-like growth factor II, macrophage inhibitory factor, and CA-125) is no longer reliable because some of the markers are not expressed timely enough to be useful for early stage detection of ovarian cancer (Mai et al., 2011).

What is recommended, on the other hand, may overlap between various histopathologic types. Initial work up of an undiagnosed pelvic mass combines laboratory studies with an abdominal or pelvic ultrasound and/or CT/MRI scan. Serum tumor markers such as CA-125, inhibin, AFP, and beta-hCG can be measured based on patient characteristics (The National Comprehensive Cancer Network [NCCN], 2018b). As an example, clinicians may consider measuring AFP levels in younger women if germ cell tumors are suspected. Furthermore, the FDA has approved HE4 and CA-125 as biomarkers to determine the risk of ovarian cancer in women with undiagnosed pelvic mass (Yoshida et al., 2016); however, measuring these biomarkers is not currently fully recommended by the NCCN and is regarded as optional by other professional organizations such as the Society of Gynecologic Oncologists (SGO) (Salani et al., 2011). If CA-125 levels were initially elevated, measures can be repeatedly taken for follow up post-treatment, but may be inconclusive in patients such as those that are asymptomatic with elevated levels of CA-125 (Lindemann et al., 2016). Others that may be useful in diagnostic tests may include the previously mentioned biomarkers, LDH and CEA (The National Comprehensive Cancer Network [NCCN], 2018b). The CA-125 to CEA ratio is occasionally taken before starting neoadjuvant chemotherapy to confirm the histology of the ovarian cancer along with biopsy results (The National Comprehensive Cancer Network [NCCN], 2018b). Family history plays a strong role in onset of ovarian cancer at an earlier age, particularly when associated with *BRCA1* and *BRCA2* genotypes (Nakonechny and Gilks, 2016); therefore, genetic analysis to identify *BRCA* mutations is important in risk-classification.

Less common histopathologies have other unique biomarkers for diagnosis and screening. In mucinous carcinoma, PAX8 immunostaining may be helpful to differentiate from adenocarcinomas that have metastasized to ovaries (Bruls et al., 2015). Endometrioid adenocarcinomas express higher levels of cytokeratin 7 (CK7), PAX8, CA-125, and estrogen receptors (McCluggage et al., 2015).

Determining an accurate sequential application of or crafting an individualized approach to diagnostic methods are some future strategies to be considered. Particularly in a tumor type with extremely diverse morphology, measuring only a serum biomarker may be insufficient and should be combined with ultrasound and CT scan (Ueland, 2017). For advancing the utility of ovarian cancer biomarkers, new types of biomarkers such as miRNAs hold promise and are also being studied extensively (Ueland, 2017). Circulating cell-free miRNAs are showing increasing potential in earlier detection in the general field of oncology (Schwarzenbach et al., 2014). A review by Nakamura et al. described the methodologies for obtaining miRNA samples and summarized studies of miRNAs with clinical relevance in ovarian cancer diagnosis, prognosis, and measuring treatment response (Nakamura et al., 2016). The ideal situation is that a combination of serum nucleic markers and imaging techniques may provide for a simplified yet comprehensive screening panel in a single test.

## Future Perspectives

In reviewing the present biomarkers in various endocrine neoplasms, several recurring themes can be noted. Firstly, measuring hormone secretion is logical and is often the initial sign that a tumor should be suspected, but it is rarely the only way to diagnose tumors of endocrine origin. Rather, a combination of biochemical, imaging, and genetic analyses, as well as patient history and presented symptoms should aid in the tumor characterization. Some of the common serum biomarkers that are used across multiple endocrine tumors are CgA (Kapoor et al., 2014; Oronsky et al., 2017), CA (Bauer et al., 2013), beta-hCG (Gilligan et al., 2010; Looijenga et al., 2014), and AFP (Gilligan et al., 2010; Looijenga et al., 2014). Current standard biomarkers and methods used are summarized in **Table 1**. Perhaps these biomarkers will further elucidate the basis of endocrinopathy. Furthermore, in the exploration for novel biomarkers, researchers are increasingly turning to miRNAs across all tumor types (Looijenga et al., 2014; Mir et al., 2016; Nakamura et al., 2016; Ueland, 2017). In the age of advanced technology and globalization, it is also important to conduct research to standardize and validate these biomarkers for universal use across the diversity of the human race.

**Table 1:** Summary of biomarkers and diagnostic tests for endocrine tumors.

Endocrine tumor origin	Biomarkers	Diagnostic tests
Thyroid carcinoma	• Thyroid-stimulating hormone (TSH) • Thyroglobulin (TG) • Calcitonin • Genetic biomarkers ∘*BRAF* ∘*RAS* ∘*RET/PTC* ∘*PAX8/PPARγ* ∘ mRNAs	• Fine-needle aspiration • Imaging ∘ (^18^F) fluorodeoxyglucose positron emission tomography (^18^FDG-PET) ∘ Gallium-68 (^68^Ga) radiolabelled somatostatin analog peptides PET/CT • Immunohistochemistry ∘ 7-gene mutation panel ∘ Gene expression classifier ∘ Galectin-3
Neuroendocrine carcinoma	• Genetic biomarkers ∘*Menin* ∘*RET* ∘ VHL ∘ JAK3 ∘ NRAS ∘ RB1 ∘ VHL1 • Ki-67 • Chromogranin A (CgA) • 5-Hydroxyindoleacetic acid (5-HIAA) • Mammalian target of rapamycin (mTOR) • CDKN1B (p27) • Circulating tumor cells (CTC) • Hormonal markers ∘ Insulin ∘ Gastrin ∘ Glucagon ∘ Somatostatin ∘ Growth hormone ∘ Calcitonin ∘ Substance P ∘ Pancreastatin	• Imaging ∘ CT and MRI ∘ Somatostatin receptor scintigraphy (SRS) ∘ ^111^Indium-diethylenetriaminepentaacetic acid (^111^In-DPTA)-octreotide scintigraphy ∘^18^F-fluorodopa and ^68^Ga-labeled somatostatin analogs PET/CT • Bloodwork to measure serum biomarkers
Pituitary carcinoma	• Ki-67 • p53 • Cytokeratin • Epithelial membrane antigen • Glial fibrillary acidic protein • CgA • hTERT • HER-2/neu • COX-2 • FGFR4 • MMP • Hormone markers ∘ Adrenocorticotropic hormone (ACTH) ∘ Prolactin (PRL) • Genetic mutational biomarkers ∘*Gsp* ∘*Ras* ∘*H-ras* ∘*Rb* ∘ Chromosome 11 deletion ∘ PTTG	• Imaging ∘^111^In-DPTA-octreotide SRS • MIB-1 staining index • Immunohistochemistry
Pancreatic adenocarcinoma	• Carcinoembryonic antigen (CEA) • Anti-oncofetal antigen • Tissue polypeptide antigen • Carbohydrate antigen (CA) 125 • CA 19-9 • TIMP1 • LRG1 • S100P	
	• Genetic mutational biomarkers ∘*p16* ∘*BRCA2* ∘ *KRAS* ∘ *TP53* ∘ *CDKN2A* ∘ *SMAD4*	• Imaging ∘ CT ∘ Endoscopic ultrasound (EUS) ∘ MRI • FNA • Immunoassays
Gonadal adenocarcinomas	Testicular cancer • Alpha-fetoprotein (AFP) • Lactate dehydrogenase (LDH) • Beta-human chorionic gonadotropin (beta-hCG) •*XIST* gene expression • miRNAs ∘ miR-371-3 ∘ miR-302/367 Ovarian cancer • CA-125 • Inhibin • AFP • Beta-hCG • HE4 • LDH • CEA • Cytokeratin 7 (CK7) PAX8 • Estrogen receptor • Genetic mutational markers ∘*BRCA1* ∘*BRCA2* miRNAs	Testicular cancer • Bloodwork to measure serum markers • PET • MRI • Ultrasound Ovarian cancer • Abdominal, pelvic, or endovaginal ultrasound • CT/MRI • PAX8 immunostaining

The complex nature of endocrine pathology requires detailed identification to differentiate an endocrine tumor from other conditions that may be suspected in the same physiologic region, or that may be metastases of other tumors (Wolin et al., 2017). As some of the endocrine malignancies are considered rare, often asymptomatic, and aggressive cancers, it is also imperative to develop biomarkers that enable earlier detection of disease. Similar to the management of other types of cancers, researchers are investigating the applicability of measuring circulating tumor cells in neuroendocrine tumors (Khan et al., 2013). New protein biomarkers have been identified for validation, such as p27 in gastroenteropancreatic neuroendocrine tumors (Kim et al., 2014), COX-2 enzyme in pituitary carcinoma (Ragel and Couldwell, 2004; Sav et al., 2012), TIMP1 and LRG1 in pancreatic adenocarcinoma (Capello et al., 2017), HMGA among others in testicular cancer (Mir et al., 2016), and various test panels recently approved by regulatory bodies for ovarian cancer (Yoshida et al., 2016).

Molecular and genetic testing combined is another strategy that may pave the way for potential uses of targeted therapies in endocrine neoplasms, which can help reduce unwanted adverse effects of systemic chemotherapy. The advent of immunotherapy is also becoming a clinical research area of interest in endocrinopathy such as thyroid carcinoma (Naoum et al., 2018). Finally, professional societies are urging clinicians and researchers to contribute to reporting programs or donate tumor samples. These collaborative efforts will facilitate the acceleration of biomarker discovery and validation, in turn advancing the care of endocrine cancer patients.

## Conclusion

The diagnosis and treatment of endocrine tumors are still challenging because of the complexity of their clinical presentations. Multiple biomarkers and modalities are required for diagnosis and management of endocrine tumors. Efforts must be warranted to develop newer biomarkers for better patient care.

## Author Contributions

YL and JH conceived the general idea. YL, HZ, and TT wrote the first draft. JH revised the manuscript. All authors read and approved the final manuscript.

## Conflict of Interest Statement

The authors declare that the research was conducted in the absence of any commercial or financial relationships that could be construed as a potential conflict of interest.
